# The Breast Impact Monitoring System: A Portable and Wearable Platform to Support Injury Prevention in Female Athletes

**DOI:** 10.3390/s25216585

**Published:** 2025-10-26

**Authors:** Cormac D. Fay, Ruby Dang, Jack Butler, Lucy Armitage, Joshua P. M. Mattock, Deirdre E. McGhee

**Affiliations:** 1 School of Medical, Indigenous and Health Sciences, Faculty of Science, Medicine and Health, University of Wollongong, Wollongong, NSW 2522, Australia; 2 Breast Research Australia, Faculty of Science, Medicine and Health, University of Wollongong, Wollongong, NSW 2522, Australia; 3 Biomechanics Research Laboratory, Faculty of Science, Medicine and Health, University of Wollongong, Wollongong, NSW 2522, Australia; 4 School of Mechanical, Materials, Mechatronic and Biomedical Engineering, University of Wollongong, Wollongong, NSW 2033, Australia; 5 School of Biomedical Engineering, University of New South Wales, Kensington, NSW 2033, Australia

**Keywords:** breast, injury, prevention, biomechanics, wearable sensor, FSR, impact, validation, protective equipment, female athletes

## Abstract

**Highlights:**

**What are the main findings?**

- A novel portable, wireless, and wearable sensing system was developed and validated for monitoring localised breast impacts in female athletes.
- The system provides reliable tackling and laboratory measurements suitable for sports injury research and protective equipment testing.

**What is the implication of the main finding?**

- Enables systematic investigation of breast injury mechanisms and the evaluation of protective strategies in women’s sport.
- Demonstrates broader potential for wearable impact monitoring in health, ergonomics, and sports biomechanics applications.

**Abstract:**

This study presents the design and preliminary validation of a novel portable, wireless, and wearable sensing system—The Breast Impact Monitoring System (BIMS)—for female athletes, developed to monitor and quantify localised mechanical impacts to the breast during high-intensity sporting activity. The platform addresses a critical gap in sports biomechanics by enabling, for the first time, objective measurement of breast forces in both controlled mechanical impact testing and preliminary on-body tackling trials for female athletes. Its application extends to advancing understanding of sports-related breast injuries, informing prevention strategies, and assessing the effectiveness of protective equipment. The BIMS leverages an array of 16 thin-film Force Sensitive Resistors (FSRs) and employs a dual-core microcontroller architecture to manage the trade-off between wireless constraints and high-speed data fidelity, successfully achieving uninterrupted acquisition at 856 Hz for each channel. The system was rigorously validated against a reference instrument using a commercial Force Plate and a custom mechanical drop rig, demonstrating high accuracy with a calibration model ($R^{2}=0.9988$). Preliminary wearable testing confirmed the system’s capability to detect and spatially map high localised impact forces, including peak forces up to 550 N (across an area diameter of 20 mm), during preliminary rugby tackling activities. By offering a practical and scalable solution for capturing previously inaccessible data, this system establishes a foundation for future research into athlete welfare and long-term breast health.

## 1. Introduction

It is well established that participation in sport, particularly at competitive or elite levels, carries an inherent risk of injury, with the high potential to adversely affect immediate performance, recovery, and long-term health outcomes [1,2,3]. Acute and repetitive trauma can result in musculoskeletal dysfunction, psychological stress, and a diminished capacity to return to sport at previous levels of competition [4,5]. Consequently, there is a sustained research effort focused on developing and validating tools, frameworks, and interventions aimed at mitigating injury risk through improved training design, load monitoring, and equipment optimisation [6].

In recent years, wearable technology has emerged as a transformative tool, offering researchers and practitioners access to real-time, in situ biomechanical [7,8], biomedical [9,10], and biochemical [11,12] information and data [13]. While laboratory-based systems—such as optical motion capture and force plates [14]—have long provided precise biomechanical measurements, they are inherently constrained by their artificial environments and often fail to capture the complex, context-specific nature of sport performance [15]. By contrast, wearable devices enable the continuous monitoring of athletes in real-world conditions, providing more relevant and accurate data [7,16]. Technological advances have driven improvements in sensor miniaturisation, battery life, and wireless data transmission, making such tools increasingly accessible and robust for field applications [15,17,18]. Despite this, the literature has historically remained disproportionately focused on male athletes, which may reflect historic participation levels, availability, or biases within sports science and elite athletic funding [19,20]. By contrast, female-specific validation and application of wearable systems remain underdeveloped [21].

This disparity is particularly salient given the rising participation of female athletes in both recreational and elite domains [22,23]. Female participation in rugby union, for example, has increased by 53.2% in 2024, and of the 8.46 million players registered worldwide, one quarter are female, narrowing the long-standing gender gap [24,25]. A similar trajectory is also observed in women’s soccer, with a 24% increase in female participation since 2019, and currently 16.6 million female players worldwide [26]. As this upward trend continues, there is a corresponding increase in exposure to gender and sport-specific injury mechanisms [27,28,29]. Yet, existing risk models and preventative strategies—often derived from male cohorts—may not adequately translate to the female population, due to fundamental anatomical, physiological, and biomechanical differences [30].

Rugby union in particular is a primary example of a high-impact and high-risk sport [31]. Due to the full-contact nature of the sport, athletes face high risks of injury, particularly during running and tackling maneuvers [32,33]. Despite accounting for a quarter of rugby union players globally, injury surveillance data in women’s rugby remains limited [34,35], highlighting continued gender inequities in sports medicine research. From the limited research, female-specific sports injuries, such as breast injuries and pelvic floor dysfunction, have been identified to significantly impact female athletes [29].

A specific and underexplored area within female sport injury research is the impact-related trauma to breast tissue [28,36]. Breast injuries are a female-specific sports injury typically caused by direct impacts to the chest, leading to pain, bruising, and swelling [28,37,38]. They can have severe long-term consequences to breast health, including breast fat necrosis, that can mimic the presentation of breast cancer, breast deformity from halting of breast development or bursting of breast implants, and damage to the breastfeeding mechanism requiring cessation of breastfeeding [39,40,41,42,43,44,45]. This is concerning considering breast cancer remains the most commonly diagnosed cancer among women globally [46].

Despite being a frequent site of injury in high-contact sports such as rugby [36], hockey [47], martial arts [48], water polo [49], amongst others [37,38,50], the breast remains underrepresented in injury surveillance and equipment testing protocols [51]. Recent investigations suggest that up to 58% of female contact sport athletes report experiencing breast trauma, though underreporting remains widespread [28,37,38]. Within female football codes—including Australian football, rugby league, and rugby union—it is estimated that less than 10% of breast injuries are reported to medical or coaching staff, despite perceived negative impacts on athletic performance (i.e., limiting the ability to run, perform forceful arm movements and sport-specific activities such as tackling) [52,53,54,55].

Minimal prevention strategies have been implemented for breast injuries within the football codes [53,54,55]. Only a minority of female athletes utilise protective breast equipment, and there is a lack of existing standardised assessments for equipment efficacy or even to measure the magnitude of breast forces involved in breast injuries to assist with equipment design [56]. Consequently, no research has been published to ensure the efficacy of currently available breast protective equipment to attenuate breast force [28,57].

Several wearable sensing approaches have been developed for monitoring chest or torso impacts in contact sports, though none are tailored specifically to breast loading. For example, microtechnology systems using accelerometers and gyroscopes have been applied in rugby to detect tackles and ruck events in match play [58,59]. An instrumented mouthguard has also been used to capture linear and rotational accelerations during collisions in elite rugby union [60]. More broadly, IMUs mounted on the chest have been reviewed for their use in activity classification, posture, and respiratory motion monitoring in wearable applications [61]. These studies confirm the viability of wearable impact sensors on the torso, yet none presently quantify localised breast force during dynamic sporting impacts. Our work therefore addresses this novel and unmet measurement gap.

The unique anatomical, size, and portability constraints of in situ breast contact force measurement during sport activities necessitate the development of a unique wearable sensor system. Wearable sensing technology that can specifically monitor mechanical impacts to the breast to measure the magnitude of the impact forces associated with breast injuries and test the efficacy of prevention strategies, such as breast protective equipment, has yet to be developed. This represents a critical gap in both sports technology and female athlete safety. In this work, we present the development and preliminary validation of a portable, wireless, wearable sensing system designed to monitor localised impacts to the breast during high-intensity sporting activities associated with breast injuries (e.g., tackling activities), which could then be used to test the efficacy of breast protective equipment. We describe the system’s design, integration, and initial testing, with the aim of establishing a foundation for future work in both injury surveillance and protective equipment evaluation for female athletes.

## 2. Materials and Methods

### 2.1. Design Requirements

The development of a breast-mounted force sensing system required careful definition of design specifications, informed by both physiological considerations and sport-specific constraints. For preliminary validation, rugby union was selected as the exemplar sport due to its high-impact nature, high prevalence of breast injuries, growing female participation, and accessibility for field trials. Rugby is widely acknowledged as one of the most physically demanding team sports, with frequent full-contact collisions, particularly during tackles, scrummages, and rucks [32,33].

Breast anatomy presents unique challenges for localised force sensing. The soft, deformable tissue composition, curvature, and inter-individual variability in size, shape, and density require the use of sensors that are flexible, planar, and conformable on the skin surface. Inspired by the planar geometry of biomedical disc electrodes commonly used in surface electromyography [62], the selected sensors needed to be soft, flexible, minimally obtrusive, low profile, and skin-compatible, capable of covering the following breast zones commonly:

- Superior Lateral Quadrant (SLQ);
- Superior Medial Quadrant (SMQ);
- Inferior Lateral Quadrant (ILQ);
- Inferior Medial Quadrant (IMQ);
- Areola and Nipple Complex (ANC).

The breast zone regions differ in dimensions due to the inter-individual variability in breast size and shape. To ensure that the sensor placements are consistent across the varying sizes and provide adequate coverage of breast tissues, the breasts are divided into the quadrant zones based on proportional distance from the superior (SB)/inferior (IB) and medial (MB)/lateral (LB) borders of the breast. Sensor placements are a proportional distance from the border lines (superior-inferior borders line = mid-line in the sagittal plane, medial-lateral borders line = transverse line in the transverse plane) that separate the breasts into quadrants and enable scaling to accommodate variations in breast sizes.

**Figure 1:**
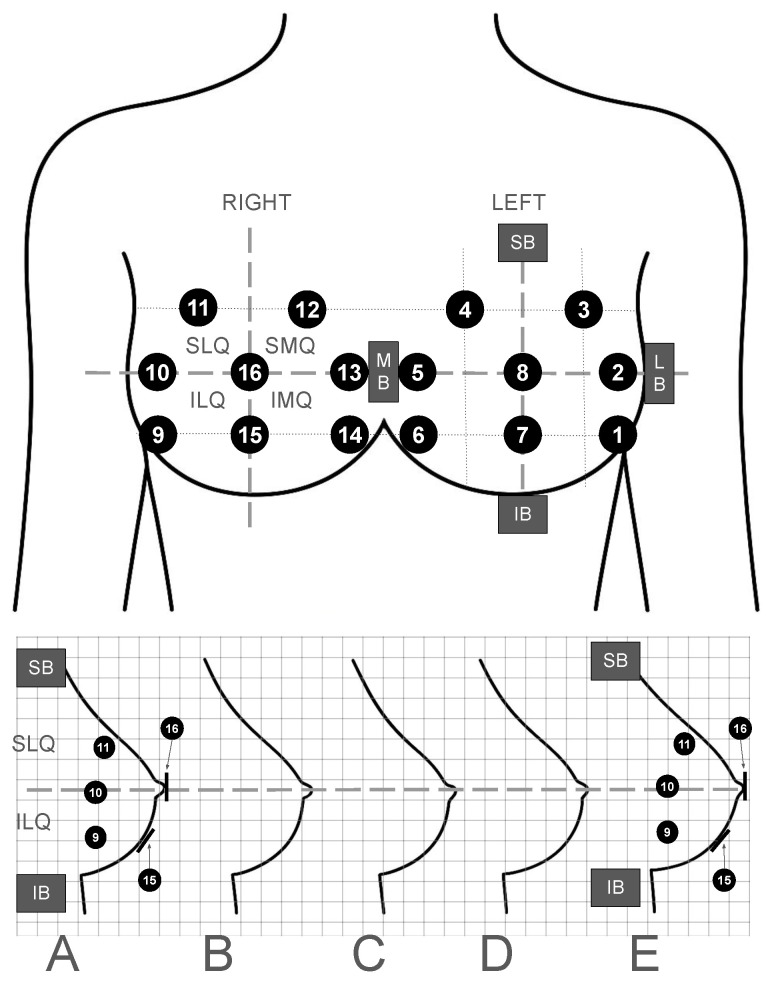
Identified locations of the sensors on the female athlete’s breasts, see Table 1 for an explanation of the sensor ID numbers. **Top**: Front view showing placements of each sensor and labelled breast quadrants. **Bottom**: Side view of the right breast of various cup sizes and visible sensors to demonstrate scale placements—sensors 15 and 16 are represented as lines as they are viewed on their side.

Figure 1 presents the chosen optimum locations for the sensors with Table 1 providing further coverage descriptions. Coverage of the primary areas was chosen because these regions are used in clinical breast examination for breast cancer, breast pain, and breast trauma (e.g., SMQ) [63]. The spatial resolution and number of sensors (16 channels in total, 8 per breast) were selected to offer a balance between anatomical coverage, data granularity, and hardware capability.

In-field usability necessitated compatibility with existing athlete garments. GPS-enabled sports bras commonly feature a small rear-mounted pocket designed to house player tracking units. This provided an ideal location for the housing of the main electronics module. The enclosure dimensions and mounting method were, therefore, constrained by the available pocket space.

### 2.2. Force Sensors

The selection of force sensors was guided by the design criteria outlined in Section 2.1, including mechanical compliance, spatial constraints, and their suitability for wearable applications, prioritising characteristics such as being light, flexible, and non-abrasive for optimal user comfort and seamless integration with undergarments. An extensive review of commercially available force-sensing technologies was conducted through electronic component distributors such as RS Components, Element14, Mouser, and DigiKey. The majority of options identified were rigid, block-type load cells or mechanically complex transducers not suitable for placement that can negatively impact soft-tissue surfaces.

Thin-film resistive sensors, particularly Force Sensitive Resistors (FSRs), were identified as the most appropriate choice based on their form factor, mechanical flexibility, ease of integration, and established use in wearable systems. To ensure suitability for the expected loading conditions, relevant literature on rugby-related contact forces was consulted. Shoulder-led tackle events and machine scrummaging data from prior studies [64,65,66] provided reference values for average and maximum loading, which informed the required sensing range.

Commercially available thin-film resistive sensors were procured from Grandado marketed (FGHGF, Motion Film Pressure Sensor, 150 g, Model AC-9V2D56QXVXV) under the description “Film Pressure Sensor Resistive Force Sensitive Plantar Flexible”. Each sensor had a nominal thickness of approximately 100 μm and a circular active sensing area 20 mm in diameter, encapsulated within a 26 mm flexible polymer film for added mechanical protection and anatomical conformity. The sensors are specified for force detection up to 150 kg, equivalent to approximately 1471 N. While the manufacturer specifies the load capacity in kilograms, all values reported here are converted to Newtons (N) to align with conventions in sports biomechanics literature.

These sensors were selected to enable direct placement on and in between layers of undergarments, while maintaining stable signal acquisition under dynamic loading conditions. The size and geometry of the sensing region were chosen to support sufficient surface area coverage across varied anatomical presentations, allowing for reproducibility and adaptation to a wide range of users.

### 2.3. Mechanical Testing System Design

To evaluate the response characteristics of the sensing system under controlled impact conditions, a custom-built vertical drop rig was designed and constructed. The aim was to replicate the magnitudes and durations of force impacts plausibly sustained by breast tissue during high-contact sports, such as rugby union, with sufficient repeatability and resolution for calibration, validation, and robustness testing.

**Figure 2:**
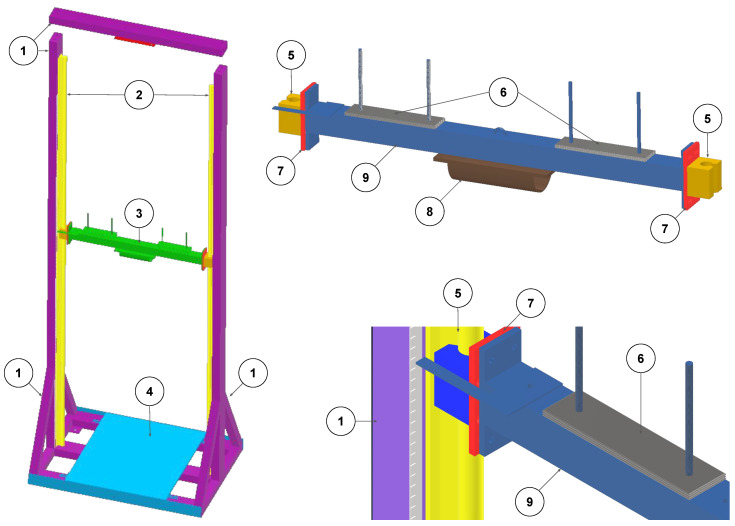
CAD drawing of rig design of force impact system—subsystems colour coded for clarity: [1] Frame (medium medium violet); [2] Rails (lime yellow); [3] Impactor (emerald green); [4] Force plate mount (sky blue); [5] Rail sliders (mustard yellow); [6] Weight mounts (dark grey); [7] Adaptor plate (scarlet red); [8] Impact surface (coffee brown); [9] Impactor housing (steel blue).

The testing rig, shown in Figure 2, comprises five key components: a rigid steel support frame (medium violet), an impactor assembly (green), dual vertical rails with low-friction sliders (yellow), a modular adaptor plate system (red), and a force plate housing (blue) for reference force measurement. The impactor is manually weighted and released from variable heights to generate repeatable impact profiles. The vertical rails originated from a CNC machine design and were selected for their low friction coefficient and good tolerance in surface straightness.

The impactor consists of a 1 kg central beam to which custom-fabricated 100 g steel plates (2 mm thick) can be added on each side. Up to 10 kg of additional mass can be mounted, bringing the total mass to 11 kg. Weight plates are secured using slide-on, quick-action nuts to facilitate efficient weight adjustment between trials. Vertical height is adjustable up to 2 m, with the drop height measured using an adhesive magnetic tape strip affixed to the rail.

A winch system (safe working load: 1134 kg) was used to raise the impactor to the target height. Release was achieved remotely via a high-strength ski hook and snap-hook assembly (rated up to 175 kg and 510 kg, respectively), with a quick-release rope pin system allowing for precise timing and safety. The release mechanism ensures free-fall conditions with minimal off-axis motion as well as a safe distance from the impact.

The target (either a rigid force plate or a breast prosthetic embedded with sensors) was mounted at the base of the rig. The reference instrument was a Kistler force plate (Model 9260AA6, Kistler Instruments AG, Winterthur, Switzerland) sampling at 10 kHz, enabling ground-truth verification of impact magnitude and duration. The plate position was adjustable via modular mounting brackets, with precautions taken to avoid edge loading or compression on load cell feet that might compromise readings.

### 2.4. Portable Device Design

**Figure 3:**
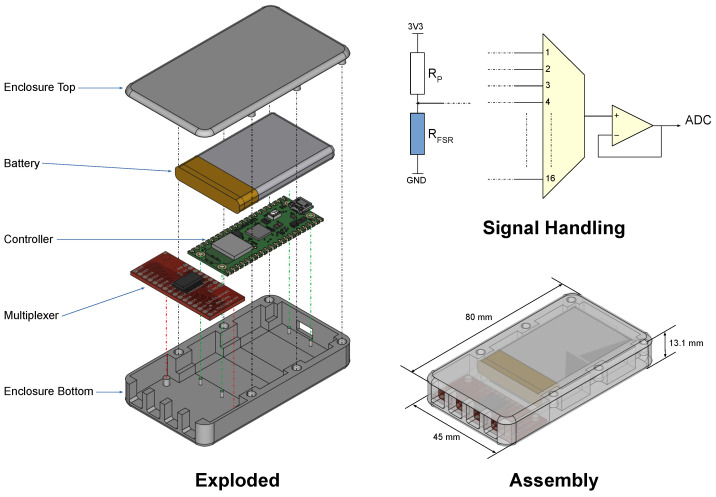
Portable platform design. The mechanics are shown in a 3D CAD drawing with the major components in exploded and assembly modes. The signal conditioning is shown (from left to right) as a potential divider(s), a multiplexer, and a buffer.

Figure 3 illustrates the mechanical and electronic design of the wireless sensing platform. Figure 3 presents an exploded view of the 3D CAD model, developed using FreeCAD (v1.0.0), to delineate the major structural components of the enclosure. The form factor was constrained by the available space within the integrated GPS pocket of a standard GPS sports bra. Internally, the base enclosure accommodates the primary components, including a 16-channel analogue multiplexer (Model CD74HC4067, Texas Instruments, Dallas, TX, USA) and a Raspberry Pi Pico W microcontroller (Raspberry Pi Foundation, Cambridge, UK), which are aligned using guide features and secured via integrated mounting posts. A 3.7 V, 1.1 Ah LiPo battery is housed above the controller, retained securely by the enclosure walls. The top cover ensures mechanical stability and retention of internal components through six press-fit inserts (three on each side).

The enclosure was fabricated using a fused deposition modelling (FDM) 3D printer (Voxelab Aquila X2) with thermoplastic filament (Polymaker PolyLite ABS Blue, Core Electronics, CE06674). Figure 3 (bottom right) shows the fully assembled platform, with the enclosure rendered semi-transparent to highlight the internal configuration. The four access points located at the base of the assembly allow for easy groupings of four for the 16 sensor wirings. Two to each side enables access to each breast, with a total of 8 locations to monitor per breast.

Figure 3 (top right) provides a schematic overview of the signal handling subsystem. Each force-sensitive resistor (FSR) is integrated into a voltage divider circuit (only one instance shown for clarity). A series resistor (RP) serves dual purposes: it completes the voltage divider and limits current flow—an important safety consideration due to the sensor’s proximity to the skin. The outputs of the voltage dividers are routed via the multiplexer, which sequentially channels each signal to a buffer stage implemented with an operational amplifier (LM358AN). This buffered signal is then fed into an analogue-to-digital converter (ADC) input on the RP2040 microcontroller for further processing.

### 2.5. Embedded System Programming

The wireless sensing platform was programmed using MicroPython v1.26 on the previously mentioned Raspberry Pi PicoW (RP2040), a low-power dual-core microcontroller with built-in Bluetooth Low Energy (BLE) connectivity. The dual-core architecture was essential in enabling the separation of high-frequency analogue signal acquisition from the lower-bandwidth wireless communication, which would otherwise act as a bottleneck during continuous multi-channel sampling.

Core 0 was dedicated to real-time data acquisition. It sequentially cycled through each of the FSRs using the analogue multiplexer (CD74HC4067), reading the output voltage from each channel via the Pico’s 12-bit ADC. Each sensor’s readings were continuously appended to its own array in memory, enabling uninterrupted sampling without delay from processing or transmission tasks.

Core 1 was responsible for data processing and wireless communication. It operated at a default rate of 1 Hz, triggered via a timed interrupt. Upon activation, this core computed summary statistics for each sensor channel, including maximum, minimum, sum total, and the number of samples collected over the previous cycle. These aggregated values were then compiled into a compact data packet suitable for BLE transmission. The raw sensor arrays were cleared following each transmission cycle to ensure fresh data collection for the next interval and to avoid buffer overloading.

This architecture allowed for efficient separation of tasks across both processing cores, addressing the limitations of BLE throughput. The decision to transmit processed rather than raw data was necessitated by BLE’s inherent bandwidth constraints, which were insufficient for real-time transmission of raw ADC data across 16 channels at high sampling rates. This compromise enabled responsive wireless communication while still preserving key signal features relevant to impact detection and temporal pattern analysis. For future implementations, on-device storage of raw data or burst transmission protocols may be considered to further enhance temporal fidelity during high-intensity events.

### 2.6. Sampling Frequency

The selection of an appropriate sampling frequency ($f_{s}$)—also referred to as Samples per second (Sps)—is critical when measuring impact dynamics, particularly for high-frequency transient events such as collisions in contact sports. The literature with respect to measurements of breast impacts is sparse or not available, especially in contact-based sports such as rugby. Therefore, the choice of a $f_{s}$ was guided by parallel studies in our target domain of rugby activities.

Usman et al. [64] mounted a custom-force plate within a tackling bag, with 4 thin-film force sensors wired to an Economical Load & Force Measurement (ELF) System (DAQ) to investigate shoulder tackles in rugby union football. The sampling frequency was reported at 1.98 kHz, which was achievable as a wired system with few sensors. As a more wearable example, Pain et al. [66] equipped a shoulder pad with a sensor matrix for measuring shoulder impacts during rugby activities. They reported a suitable sampling frequency of 250 Hz for impact measurements. Preatoni et al. [65] reported a system for measuring the forces generated during machine scrummaging in rugby and capable of measuring at 500 Hz, detailing the ability to measure peak forces.

In contrast to the above examples that focused primarily (and understandably) on shoulder impact forces (a relatively rigid anatomical site supported directly by the underlying scapula), the breast is expected to deform more extensively upon impact due to its soft tissue composition. Given that prior literature has established that the duration of impact increases with the deformability of the impacted surface [67], it is reasonable to anticipate longer impact durations in breast impacts compared to previously studied anatomical sites such as shoulders. A suitable analogy would be a person jumping on pavement as opposed to a trampoline. Therefore, operational expectation was 250–500 Hz based on previous studies involving rugby; however, a slower sampling frequency could suffice given that the breast is a deformable ‘surface’.

Considering the importance of sampling frequency for this application, we investigated the capabilities of our platform through a number of preliminary tests whereby the sampling frequency was varied. This provided an informative choice of how many sensors to place on each breast—balancing the capabilities of our system and the required sensing locations.

### 2.7. System Calibration

The calibration of the developed breast impact monitoring system was crucial in evaluating its response characteristics and ensuring its accuracy and reliability. This process involved both direct calibration of the sensing elements and verification of the system’s performance against a high-fidelity reference instrument. The response of the developed system (ADC response) was assessed as a function of the known applied impact forces. This was achieved by applying controlled forces using the drop rig, with the force plate placed beneath, and extracting the maximum value of the resulting profile. Please note that while the biomechanics literature typically reports impact magnitudes in terms of net force (N), it is important to note that the system’s FSRs fundamentally capture localised pressure (Pa) at discrete skin-sensor interfaces. Each FSR has an active sensing area of 20 mm in diameter (∼3.14 $\times \;10^{-4}\;m^{2}$), enabling the quantification of surface pressure at anatomically specific regions of the breast. This localised pressure is then correlated to an applied force for contextual comparison with existing literature.

### 2.8. Data Flow and Analysis

The data acquisition and analysis pipeline was designed to accommodate high-frequency force inputs while supporting real-time wireless transmission and comprehensive post-session analysis. The embedded logic employed a dual-core architecture on the Raspberry Pi Pico W microcontroller:

- Core 0 (Acquisition): Dedicated to real-time data capture from 16 force-sensing resistor (FSR) channels via the 12-bit ADC at a composite sampling rate of 856 Hz. Raw data were continuously buffered in local memory, ensuring uninterrupted sampling without interference from processing or communication tasks.
- Core 1 (Processing and Transmission): Operated on a timed interrupt at 1 Hz to process the incoming data stream. For each channel, the core computed summary statistics (maximum, minimum, sum total, and sample count) and transmitted these via Bluetooth Low Energy (BLE) to a client laptop. This design reflects a deliberate trade-off between temporal resolution and bandwidth limitations, as BLE could not support continuous streaming of 16 channels at the full sampling rate. To ensure data integrity and enable comprehensive offline analysis, the complete raw dataset from each session was stored locally on the device.

For post-capture analysis and visualisation, subsequent processing and visualisation were performed in Python 3.11.13 using standard scientific packages (numpy [68], scipy [69], and matplotlib [70]). The discrete force data from each sensor were first converted to Newtons using the calibration model (Section 2.7). The low relative standard deviation (mean RSD = 1.48%) observed across calibration points confirms the precision and stability of the measurements.

Transmitting summary statistics at 1 Hz was therefore considered an acceptable compromise: Core 0 maintained continuous high-frequency capture to preserve the fidelity of short-duration impacts, while Core 1 provided low-bandwidth, real-time monitoring through aggregated features. The strong exponential relationship between the reference force plate (x-axis) and the BIMS response (y-axis) demonstrates that, even at reduced reporting rates, the system accurately reproduces the magnitude of applied forces.

To visualise spatial force distribution across the breast surface, force magnitudes from the eight sensors on each side were interpolated over a two-dimensional grid representing the breast contour. Sensor coordinates were mapped to an image reference frame, and instantaneous forces were interpolated using a Gaussian-weighted radial basis function (RBF), implemented via the scipy.interpolate module. Each sensor contributed a spatial weighting defined by Equation (1), where the influence decayed with distance from its position, and σ was proportional to the average inter-sensor spacing. The interpolated field was then masked to the breast contour and colour-mapped to generate continuous heat maps, providing a smooth and intuitive representation of localised loading without inferring additional measurement resolution beyond the physical sensor array. Videos were generated for an accessible spatial and temporal analysis of the laboratory trials.

(1) $$\omega_{i}\left(x,y\right)=F_{i}\cdot \exp\left(-0.5{\left(d_{i}/\sigma \right)}^{2}\right)$$

### 2.9. Preliminary Testing and Wearability Testing

The last stage of testing involved wearability testing, where the device was worn by 4 female participants with various breast sizes. All 4 engaged in comfort and wearability, while preliminary data was collected from two participants as they had football codes experience. Sensors were placed on top of the participant’s bra and secured using transparent medical tape. A GPS crop top was worn over the sensors to contain the Bluetooth battery of the sensors; it also assisted in holding them in place.

Simulated rugby-related tackling activities involved participants each performing over the ball and under the ball active shoulder tackles. Each set of tackles included 4 tackle repetitions with approximately 30–60 s rest in between each tackle. Participants alternated between the role of ball carrier and tackler, and were instructed to begin 6 m apart and perform the tackle event at 80% match intensity.

All procedures were conducted under institutional ethical approval (Ethics Number 2024/048), and data collection adhered to approved participant consent procedures, ensuring privacy and comfort. The Bluetooth Low Energy (BLE) transmission used by the sensors emits signals of negligible intensity, substantially lower than conventional Bluetooth communication, thereby posing no safety or interference risk to participants. Furthermore, data were transmitted at a low sampling rate (1 Hz) during preliminary testing, which further minimises any potential exposure and power demand. All measurements were collected respectfully, in a secure, private laboratory with only female researchers present. The sensors were positioned over the participants’ own sports bras, and no direct contact with the skin was required.

## 3. Results and Discussion

### 3.1. System Realisation

**Figure 4:**
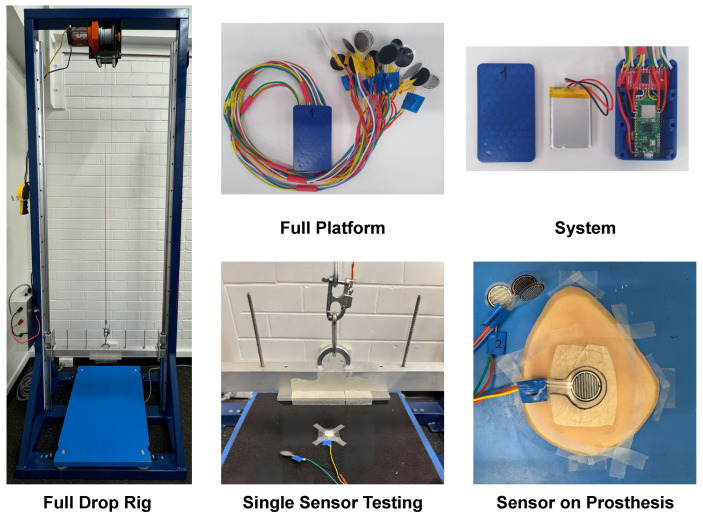
Captured image of the developed system. Left: Drop rig. Top centre: Wireless platform with equipped FSR sensors. Top right: Platform with major visible components. Bottom centre: Placement of a sensor on the force plate and under the impactor. Bottom right: Placement of a sensor on a prosthetic breast.

The comprehensive development of the Breast Impact Monitoring System (BIMS) involved the fabrication of both a custom mechanical testing rig and the portable wireless sensing platform itself. Figure 4 provides a visual overview of the realised system components. The custom-built vertical drop rig, designed to generate repeatable and controlled impact conditions, consists of a rigid steel support frame, an impactor assembly with adjustable mass (up to 11 kg total) and drop height (up to 2 m), dual vertical rails for low-friction guidance, a modular adaptor plate system, and a force plate housing for ground-truth reference measurements. The impactor release mechanism ensures free-fall conditions with minimal off-axis motion, enhancing experimental precision and safety. This setup enables testing across a broad range of impact forces, up to approximately 13.8 kN, depending on the selected mass, drop height, and surface compliance, allowing for a realistic simulation of dynamic loading conditions experienced in human motion.

The core of the wearable system, termed the “Full Platform”, comprises the thin-film Force Sensitive Resistors (FSRs) and the enclosed electronics module. These FSR sensors, approximately 100 μm thick with a 20 mm diameter active sensing area encapsulated within a 26 mm flexible polymer film, were selected for their mechanical flexibility and suitability for soft-tissue interfaces. A total of 16 channels are employed, with 8 sensors designated for each breast to ensure comprehensive coverage across the superior lateral (SLQ), superior medial (SMQ), inferior lateral (ILQ), and inferior medial (IMQ) quadrants, and the areola and nipple complex (ANC).

The electronics module, designed for integration into the rear pocket of a standard GPS sports bra, houses a 16-channel multiplexer and a Raspberry Pi PicoW microcontroller, powered by a 3.7 V, 1100 mAh LiPo battery. This enclosure was fabricated using fused deposition modelling (FDM) 3D printing. As illustrated in the “Electronics” view of Figure 4, the internal configuration includes the controller, multiplexer, and battery, secured within the enclosure. The system’s signal handling subsystem incorporates each FSR into a voltage divider circuit, with signals routed through the multiplexer to a buffer stage and then to the microcontroller’s analogue-to-digital converter (ADC) for processing. The system’s capability to monitor localised force at discrete skin-sensor interfaces allows for detailed spatial mapping of impact distributions across breast tissue, offering unique insights into localised loading patterns. The “Single Sensor Testing” and “Sensor on Prosthesis” images in Figure 4 further demonstrate the methodological setup for calibrating and validating the FSR sensors under controlled conditions, including their placement on a force plate and a prosthetic breast for mechanical evaluation.

### 3.2. Force Sensors and Magnitude

The decision to employ thin-film Force Sensitive Resistors (FSRs) in the system was driven not only by their mechanical flexibility and low-profile geometry but also by their proven effectiveness in wearable technologies involving soft-tissue contact and dynamic loading. Previous studies have demonstrated the suitability of FSRs in human–machine interaction, gait monitoring, and sports applications [71,72,73], highlighting their adaptability to variable anatomical surfaces and movement conditions. In particular, Usman et al. [64] successfully applied thin-film sensors in rugby tackling contexts, providing a domain-specific precedent that supports the current implementation.

While no direct data exists on the magnitude of forces experienced by breast tissue during contact sport, related upper body measurements provide a relevant reference range. Usman et al. [64] reported shoulder contact forces between 500 N and 3800 N during over 700 tackle events, using a four-sensor array. Preatoni et al. [65] estimated machine scrummaging forces among female rugby athletes at approximately 8.7 kN (group total), while individual interactions with scrum machines by male players have generated peak forces in the range of 1–2 kN [74]. Similarly, Pain et al. [66] recorded tackle collisions reaching up to 2.8 kN.

Although these values reflect forces transmitted through the upper torso rather than directly through the breast, they provide plausible upper-bound estimates due to anatomical proximity and shared inertial pathways. In practice, the forces directly experienced by breast tissue are expected to be lower; however, in vivo quantification remains limited, necessitating conservative sensor selection.

Insights from clinical mammography provide useful context on forces tolerated by breast tissue under direct compression. Standard mammography involves applying roughly 100–200 N of compression force to achieve sufficient tissue flattening for optimal imaging [75,76,77]. Within the U.S., regulated by the Mammography Quality Standards Act, mandated compression ranges between 111 and 200 N [78]. Observational studies capture discomfort dynamics under these loading conditions—one Amsterdam-based study (n = 117) recorded peak pain ratings during the immobilization (clamping) phase at ±1500 N, with average forces ranging similarly throughout the cycle [79]. Meanwhile, pain is reported on a 10-point scale (0–10 NRS), often reaching moderate to severe intensity (NRS ≥ 4–7) when compression exceeds 100–150 N. Moreover, pressure-standardized protocols (10 kPa target pressure) have reduced reported pain while maintaining imaging quality, suggesting that both force magnitude and application method are critical in mediating discomfort [80].

These studies of force ranges in the clinical and sporting settings provide a conservative upper bound and reinforce the appropriateness of our FSR sensors’ target detection range (1000 N) for capturing relevant tissue-loading events. The specific FSRs selected were also evaluated for geometric suitability. The 20 mm sensing diameter was chosen to balance spatial resolution with anatomical coverage, enabling placement across key impact regions while preserving comfort and conformance. This sizing strategy acknowledged the variability in breast shape and size across the female athletic population and aimed to ensure adequate sensing across a representative anatomical range. Placement flexibility and sensor modularity were thus prioritised to support the eventual development of scalable or adaptive sensor configurations for broader applications.

### 3.3. Verification of Sampling Frequency

**Figure 5:**
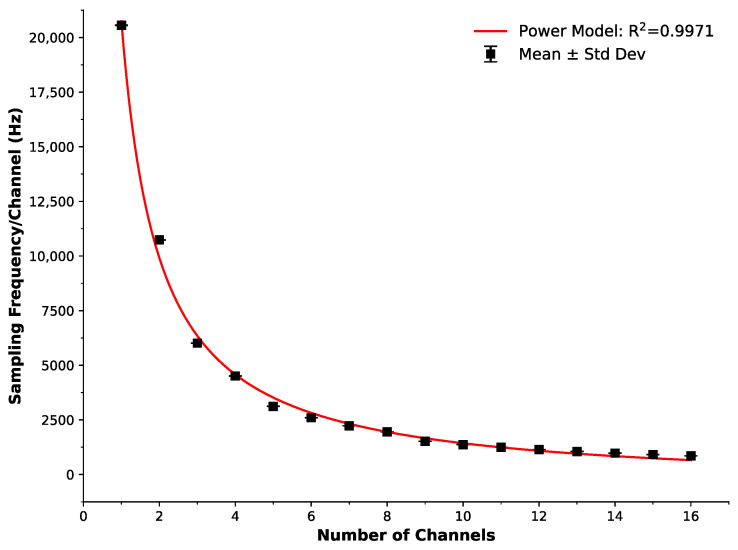
Sampling frequency capability of our portable system with respect to the number of employed channels. The data points represent an average of 60 s of testing, with the error bars as the standard deviation. The red line is a power model ($y=a\cdot x^{-b}+c$) with an excellent fit to the data (R^2^ = 0.997).

The sampling frequency capability of our portable system with respect to the number of measured channels is displayed in Figure 5. The data points represent an average of 60 s of testing, with the error bars as the standard deviation. The red line is a power model ($y=a\cdot x^{-b}+c$) with an excellent fit to the data (R^2^ = 0.9971).

Figure 5 presents the $f_{s}$ capability of our system from sampling 1-channel at 20.6 kHz, to analysing each of the 16 channels at 856 Hz ($T_{s}$ = 1.168 ms). A power model ($y=a\cdot x^{-b}+c$, red line) was applied to the measured data points (black squares), resulting in an excellent fit to the data (R^2^ = 0.9971). While this sampling frequency is lower than typical high-end force plates (often sampling in the kHz range, e.g., Usman at 1.98 kHz), it must be noted that force plates typically measure a single channel (in comparison to our system at almost 20.6 kHz). In comparison to the reported systems in the literature, our sampling frequency of 856 Hz per 16 channels represents a significant improvement; for example, Pain et al. [66] at 250 Hz and Preatoni et al. [65] at 500 Hz.

Based on our findings above, we chose to implement all 16 channels to allow impact forces in each breast region, as outlined previously in design requirements (i.e., 8 per breast), to be individually investigated and quantified. The chosen 16 channels were an effective balance between breast coverage and sampling frequency established by previous research. Sufficient breast coverage is required to investigate breast injuries in contact sports because any area of the breast could be impacted. While this may be considered in excess for smaller breast sizes, it also enables scalability for larger sizes. It must also be noted that previous studies have had wired systems in place and, therefore, relatively limited portability capabilities. Our system not only exceeds Pain’s and Preatoni’s sampling capabilities, but it also enables a wireless system (unattached) capable of monitoring participants during play, and its small form factor complies with standard GPS pocket tops, enabling seamless adoption into sporting activities. Please note that all 16 sensor channels were sampled via a single 12-bit ADC (ADS1115) through a 16-channel multiplexer (CD74HC4067), managed by Core 0 of the Raspberry Pi Pico W microcontroller. Channels were read sequentially at a composite rate of 856 Hz per channel (total throughput ≈ 13.7 kHz), resulting in quasi-synchronised acquisition with a full cycle time of approximately 1.17 ms.

**Figure 6:**
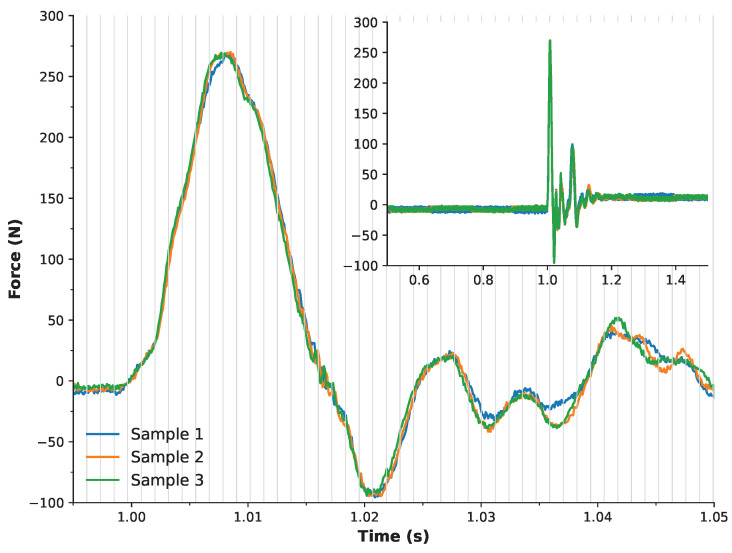
Force plate recordings following the drop of a ∼300 N load, performed in triplicate. The main plot shows time-aligned force signals, with alignment at t = 1 s based on a threshold defined as the mean plus five times the standard deviation of baseline noise. This view shows 55 ms—spanning from 0.995 s to 1.05 s, highlighting the precise moment of impact. The vertical grey lines represent a possible sampling frequency of 856 Hz as per a 16-channel implementation. The inset provides a broader temporal context, displaying data from 0.5 s to 1.5 s.

While the system’s sampling capabilities have been compared to values reported in the literature, it was also important to verify performance against a reference instrument. To this end, a ∼300 N weight was applied to the custom rig (Figure 4), and drop tests were conducted on a force plate sampling at 10 kHz. This procedure was repeated in triplicate. Figure 6 presents the recorded responses from all three trials, with the signals time-aligned at t = 1 s. Alignment was achieved using a threshold method based on the mean plus five times the standard deviation of the baseline signal. The overlaid waveforms show excellent agreement, indicating high reproducibility of the setup and procedure. An inset in the figure provides a broader temporal view (0.5–1.5 s) for context. Most notably, vertical grey bars indicate the potential sampling interval of the developed system when operating across all 16 channels (856 Hz). The main impact peak is captured within approximately three of these intervals, suggesting that the system’s effective sampling rate is sufficient to preserve the critical features of the event.

### 3.4. Impact Testing and Calibration

**Figure 7:**
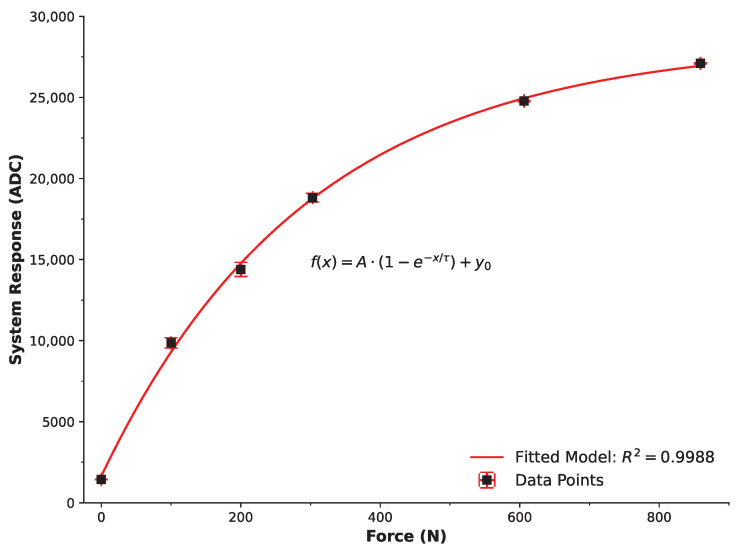
System Calibration of the Breast Impact Monitoring System. Data points represent the average of multiple max points (e.g., Figure 6) as a function of known impact forces detected by the developed BIMS system. The trend conforms to an exponential model $f\left(x\right)=A\cdot \left(1-e^{-x/\tau }\right)+y_{0}$ with an excellent fit (R^2^ = 0.9988).

The system’s calibration and preliminary testing were conducted to evaluate the response characteristics of the sensing system under controlled impact conditions. The calibration process involved assessing the developed system’s response as a function of known applied forces (Figure 7). These data points represent the maximum impact force subjected to the FSR sensors. The trend of the data suggested an exponential rise, with $f\left(x\right)=A\cdot \left(1-e^{-x/\tau }\right)+y_{0}$ offering the best description and an excellent fitted model resulting in R^2^ = 0.9988. This calibration model enabled the conversion of the system’s response (in ADC values) to known impact forces and, therefore, was necessary for determining impact forces to the breast during validation trials.

It is essential to acknowledge the inherent operational characteristics of FSRs as reported in the literature. While the relationship between force and resistance in FSRs is non-linear, the excellent fit achieved by the exponential calibration model ($R^{2}=0.9988$) confirms that this non-linearity is accurately mapped within the relevant operating range. Furthermore, FSRs can exhibit hysteresis or drift under sustained static loading. In this study, the BIMS is designed exclusively for high-frequency, transient impact detection, where sensor loading durations are in the millisecond range. Consequently, time-dependent drift effects are negligible and do not influence peak force detection. Calibration testing also demonstrated high reproducibility, with a mean relative standard deviation (RSD) of 1.48% across calibration points, indicating minimal variability and confirming the reliability of the FSR response. Regarding high-pressure response, the FSRs used are rated to 1.47 kN, while calibration and validation testing were conducted below approximately 900 N and 550 N, respectively—well within the sensors’ linear-exponential operational envelope. We therefore maintain high confidence in the calibration accuracy, stability, and repeatability of the recorded measurements under conditions representative of contact-sport impacts.

The successful calibration and verification of the system in the controlled mechanical system established its accuracy and repeatability against a known reference. This foundational laboratory validation was a prerequisite for wearable deployment. The subsequent phase assessed the system’s performance, comfort, and ability to record dynamic, non-repeatable impact forces during simulated rugby tackling, thereby extending validation beyond the limitations of a purely mechanical context.

### 3.5. Preliminary Wearable Testing

**Figure 8:**
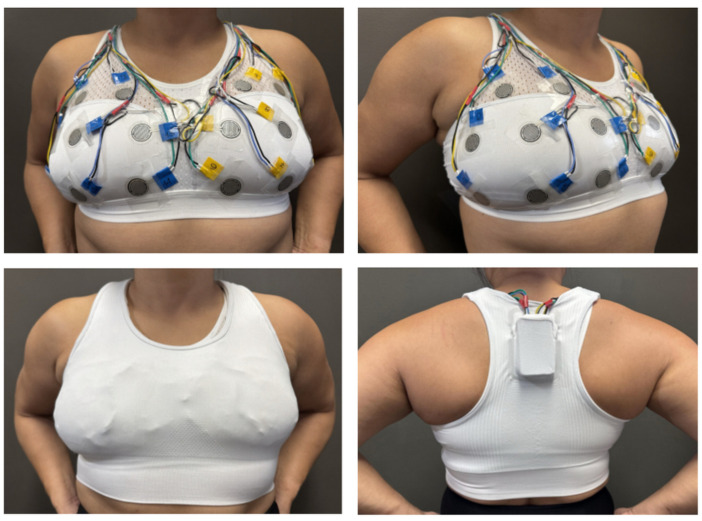
Captured images of the developed system on a participant. Top left: Front-on view of a participant wearing the developed system. Top right: Side-on view of a participant wearing the developed system. Bottom left: Participant wearing a GPS bra over the top of the developed system. Bottom right: Developed system enclosure located within the posterior pocket of the GPS bra.

The final phase of system validation involved preliminary wearability testing to assess the device’s practical application and user acceptance in a simulated sporting environment. The device was evaluated by four female participants, with preliminary data collection performed on two of these individuals, (Participants 3 and 4, as they have previous rugby experience). Table 2 presents the participants’ characteristics. Figure 8 presents a visual depiction of the developed system being worn by a participant.

During these tests, the FSRs were strategically positioned directly onto the participant’s existing bra in the positions outlined previously in Section 2.1 and illustrated in Figure 1. To ensure that the sensors remained securely in place and to further integrate the system discreetly, transparent medical tape was utilised. A standard GPS crop top was then worn over the entire sensor array. This outer garment served a dual purpose: it contained the electronic module, which was housed within the GPS bra’s rear pocket, and it provided additional support to maintain sensor placement during dynamic movements. Figure 8 (bottom right) shows the developed system (shown previously in Figure 4) enclosure placement positioned within the posterior pocket of the GPS bra, while the top images illustrate the system’s appearance on a participant from front and side perspectives.

As described previously in Section 2.9, wearability testing was conducted and subsequently investigated. Feedback from all participants indicated that the device was innocuous, non-restrictive, and comfortable to wear throughout various movements, including running, forceful arm movements (such as passing a ball or engaging in rugby-related contact), and the simulated tackling activities described in the next section. The impact magnitudes applied during preliminary testing were intentionally submaximal (up to approximately 300 N, representing 80% of the predicted maximal safe load) to ensure participant safety during initial validation. Although these loads were lower than those typically observed in competitive collisions, the calibration curve demonstrated a consistent linear response up to 900 N, and the sensors themselves are rated to withstand forces up to 1471.5 N. This confirms that the system hardware and data-acquisition pipeline can accurately capture higher-magnitude impacts beyond those tested here. Subsequent work will therefore extend testing to high-intensity impacts using instrumented manikins and field-based trials.

It is acknowledged that the small sample size used in this preliminary evaluation (n = 4 for wearability assessment; n = 2 for impact data collection) limits the generalisability of findings to the broader female athletic population. This phase of research was designed as a proof-of-concept validation to demonstrate the technical feasibility, comfort, and performance of the BIMS, rather than to establish population-level statistical inference. Importantly, the inclusion of participants with different breast sizes and garment configurations was intentional, allowing assessment of the device’s adaptability to varied morphologies and fit conditions. The consistently positive participant feedback on comfort, freedom of movement, and sensor stability indicates that the prototype design is suitable across these variations. Future work will extend this validation to larger and more demographically diverse athlete cohorts, incorporating in-field testing during live training or competitive play to confirm sensor reliability, comfort, and data reproducibility under realistic sporting conditions.

### 3.6. Preliminary Tackling Activity Testing

Considering that rugby-related tackling is considered the leading cause of injury in both rugby union (15 s) and sevens, and differences in technical execution (including tackle height and body position) have a substantial influence on performance outcomes and injury risk [82]. More specifically, rugby tackling encompasses a variety of techniques, with both over-the-ball and under-the-ball approaches identified within performance and injury risk analyses in both rugby union and rugby league contexts [83]. In terms of female rugby players, these activities can present an injury risk to their breasts and, as such, the over-the-ball and under-the-ball approaches were chosen to further validate the developed system, with methodology described previously in Section 2.9.

**Figure 9:**
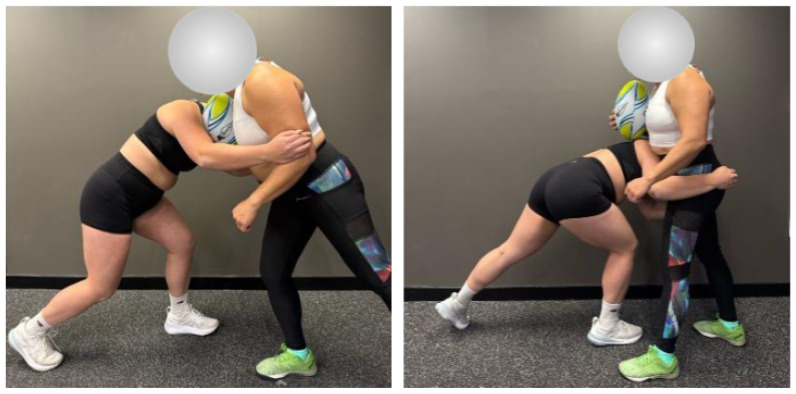
Captured images of laboratory-based rugby-related tackling activity. **Left**: Over-the-ball active right-shoulder tackle. **Right**: Under-the-ball active right-shoulder tackle.

Figure 9 presents captured images of the two participants demonstrating both over-the-ball and under-the-ball active right-shoulder tackles. These images illustrate the typical body positioning and contact mechanics involved in each tackle variation. The over-the-ball tackle (left) shows the tackler targeting the upper torso of the ball carrier, while the under-the-ball tackle (right) demonstrates a lower point of contact, aimed beneath the ball to destabilise the opponent. As discussed in Section 2.9, these simulated tackles were performed at 80% match intensity from a 6-m approach, approximating realistic in-game scenarios under controlled testing conditions.

#### 3.6.1. Over-the-Ball Tackling

**Figure 10:**
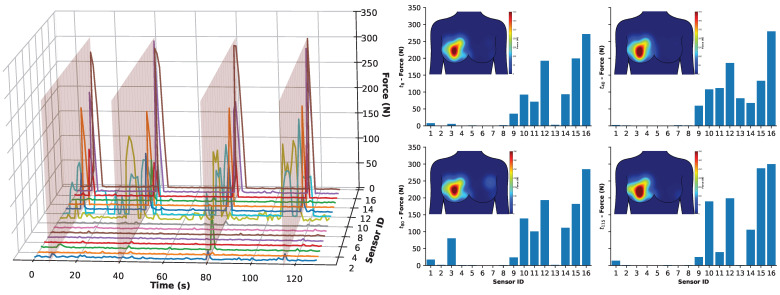
Analysis of the over-the-ball rugby activity on the receiving participant registering localised forces per sensor. **Left**: Time series of all 16 sensors shown as colored lines with transparent red planes highlighting when peak activity was detected at times: 8, 40, 80, and 115 s. **Right**: Bar chart at each of the four times with associated heat maps (top left) showing the distribution of the force across the breast, and a colour legend from 0–350 N is associated with the heat map of the force distribution. See Video S1 in the ESI.

Figure 10 provides an analysis of the over-the-ball rugby activity on the receiving participant, registering localised forces for each of the 16 sensors. Specifically, the figure is composed of two main visualisations of the sensors, i.e., time series (left plot) and Bar charts with heat maps (right plots).

The left plot presents a time series of all sensor responses over the duration of the trial. While viewing all 16 channels in a single plot can be difficult to visualise, the data remains informative. Firstly, this plot shows four distinct times when sensors were activated, corresponding with the times of tackling. Secondly, when investigating which sensors respond, it can be seen that sensors 1–8 (left breast) remained relatively inactive, while sensors 9–16 (right breast) were triggered, highlighting the system’s capability to detect localised impact with minimal/no crossover when worn by a participant. Thirdly, this enabled the automatic detection of four tackling times using integrated analysis, which are highlighted using transparent red planes overlaid on the data at approximately 8, 40, 80, and 115 s.

The right plot holds four-column plots showing data extracted at each of the four detected peak impact times (t = 8 s, t = 40 s, t = 80 s, t = 115 s). Each chart is accompanied by an associated heat map (located in the top-left corner of each subfigure) that visually represents the distribution of force across the breast, along with a corresponding colour legend. These visualisations offer a more intuitive interpretation of the force distribution across the sensor array. Additionally, an animated view of these force distributions across the breasts for the trial period is available in Video S1 (see the Supplementary Materials).

The data presented in Figure 10 suggests that the participant consistently held the ball in the same position, centrally over the areola (which corresponds to sensor 8, as identified in Table 1 and Figure 1). During impact events, the ball was pressed against the participant’s right breast, leading to the activation of sensors 9–13. This activation indicates temporary deformation of the breast tissue and confirms the system’s capability of responding to localised pressure changes.

Furthermore, the data shows that the breast experienced approximately 300 N of impact force during these controlled, relatively low-intensity tackles. It is, however, important to note that impacts during real-world tackles can be significantly higher than this. These results nonetheless demonstrate the system’s strong potential to monitor breast forces in a non-intrusive, wireless, and portable manner.

#### 3.6.2. Under-the-Ball Tackling

**Figure 11:**
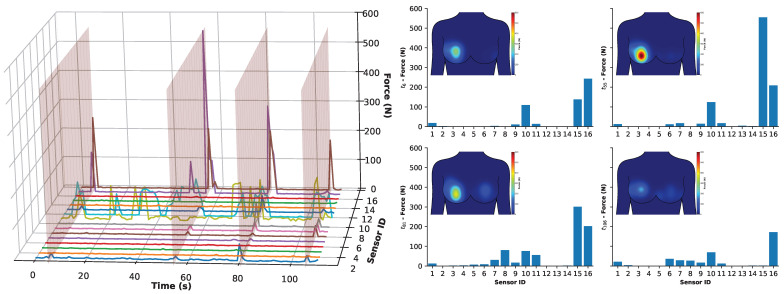
Analysis of the under-the-ball rugby activity on the receiving participant registering localised forces per sensor. **Left**: Time series of all 16 sensors shown as colored lines with transparent red planes highlighting when peak activity was detected at times: 6, 55, 81, and 106 s. **Right**: Bar chart at each of the four times with associated heat maps (top left) showing the distribution of the force across the breast, and a colour legend from 0–600 N is associated with the heat map of the force distribution. See Video S2 in the ESI.

Figure 11 presents the corresponding data when analysing the under-the-ball tackling sensor data. The data is presented in the same format as before, with Figure 11 for consistency. The time series in the left figure enabled the detection of the four primary activity times and signified using transparent red planes at 6, 55, 81, and 106 s.

The column and heat maps on the right of Figure 11 provide a more accessible view of the tackling periods. Here, it can be seen that once again the left breast sensors are relatively less responsive than the right breast, as this is the breast under load during under-the-ball tackling, as seen in Figure 9. The data also suggests that the primary responsive sensors are located at sensors 15 and 16, i.e., the areola and between the interior quadrants of the right breast, which corresponds to where the ball was held by the receiver as seen in Figure 9. Given that these are the primary two sensors, in addition to others, more prominently sensor 10 (between superior and inferior lateral quadrant), it suggests that the primary impact was between 15 and 16, with a lesser response to 10, indicating that the ball was also held laterally.

Importantly, the force impacting the breast can range from ca. 200–300 N, with one peak (t = 55) demonstrating a significant peak force of 550 N. This was a result of the second tackle being more intense on sensor 15, i.e., the force from the tackler was introduced from below between the inferior quadrants. This represents a change in intensity and shows a challenge in reproducing exact tackling behaviour. While this would be interesting to investigate in the future, it is beyond the scope of this study in demonstrating the capabilities of the wearable sensing system.

It is interesting to note that the pattern emerging from the sensor activation may be able to categorise certain patterns reflecting physical activity. This may aid medical-based injury treatment, where a female player may suffer an injury and inform medical practitioners to enable recovery.

### 3.7. Significance of Detected Localised Forces on the Breast

The preliminary tackling activities in this study recorded localised impact forces in the range of approximately 300–550 N over a sensor area of 3.14 cm^2^ (20 mm diameter). This equates to localised pressures approaching 1 MPa, which—though below bone fracture thresholds—are substantial for soft, unsupported tissues such as the breast. These forces were detected under submaximal tackling intensity, suggesting that equivalent or greater loads may be common under full-contact match conditions.

While previous studies of rugby-related contacts using instrumented tackle bags and force plates report whole-body forces between 500–3800 N during shoulder-led collisions [54,64,66], those values are distributed across much broader contact areas, typically involving the upper torso or shoulder. By contrast, the present system records force magnitudes over the localised sensor area only. This means that although the absolute pressure distribution across the breast surface cannot be determined, the detected forces still represent highly concentrated loading when interpreted relative to the sensor footprint. Such loads are consistent with contact from small anatomical features—such as the tip of a shoulder, elbow, or forearm—that frequently drive direct pressure into the chest region during tackles. For this reason, reporting forces rather than inferred pressures may be a more meaningful approach for interpreting localised breast loading.

Importantly, localised forces in the 300–550 N range fall within levels known to be capable of producing soft tissue trauma, particularly when delivered rapidly and repeatedly. Blunt-force trauma to the breast can result in pain, bruising, hematoma formation, and fat necrosis, which may calcify and be misinterpreted as malignancy in later medical imaging [38,50]. Fat necrosis and post-traumatic lump formation are documented complications in both clinical breast injury cases and sports-related incidents [84]. Yet despite the prevalence of such outcomes, breast injuries remain highly under-recognised in contact sports, with studies reporting that 48% of female collegiate athletes self-reported a breast injury, but fewer than 10% sought medical attention [38].

In women’s rugby specifically, up to 64% of players report experiencing contact breast injuries (CBIs) annually, often occurring during tackles and frequently resulting in persistent pain, swelling, or reduced performance—despite continued participation [36,84]. These injuries are rarely acknowledged in protective equipment design, training guidance, or return-to-play protocols.

To anchor these findings in more familiar contexts, such as mammography, which is a common diagnostic procedure, typically applies 200 N of compressive force per breast [85] or higher at 300 N using manual compression [86]. However, these loads are distributed across a much larger area (typically 100–200 cm^2^) than the 3.14 cm^2^ sensor sites in this study. While our system quantifies force directly at the sensor locations (and the true load may in practice be spread over a broader region of the breast), the detected forces nevertheless indicate that highly concentrated impacts can occur under dynamic sporting conditions, in contrast to the controlled loading of medical imaging.

From a structural standpoint, the fibro-adipose structure of the female breast is formed by complex scaffolding, consisting of layers of fibrous tissue pockets embedded with adipose tissue, which is firmly attached to the perimeter of the breast [87,88]. The fibroadipose tissue surrounds and protects the delicate corpus mamma that is embedded within it. The fibrous tissue is composed of collagen, which is viscoelastic in nature and prone to stretching or microtrauma during rapid loading (strain-rate dependency). The adipose tissue is soft and deformable and firmly encased within the fibrous pockets. Excessive compression and shear force to the breast can result in contusion of the fibroadipose tissue and also potentially damage the glandular tissue (corpus mamma) that it protects. Research from motor vehicle accidents has found contusion of the fibroadipose tissue can result in breast fat necrosis [39,89], with case study data also finding compression and shear forces can result in tearing of the ducts within the corpus mamma in lactating women [42].

While this analysis provides valuable insight into localised loading magnitudes, it is also recognised that the breast exhibits complex, heterogeneous mechanical behaviour. The breast is a composite, deformable structure comprising skin, adipose, glandular, and fibrous connective tissues whose thickness and stiffness vary both within and between individuals. Factors such as age, hormonal state, body mass index, and breast size influence these local properties, as does the proximity of the sensor site to the underlying chest wall. Consequently, identical external impact forces may generate different local pressure–time responses depending on anatomical location and tissue composition.

The present platform was intentionally designed to isolate and characterise loading in a direction normal to the sensor impact loading under controlled laboratory conditions to ensure calibration accuracy and repeatability. However, in authentic sporting environments, breast deformation and impact vectors are multidirectional, involving complex shear and combined normal and shear loading/components. Establishing the Breast Impact Monitoring System (BIMS), therefore, represents a crucial first step that now enables systematic exploration of these factors in future studies. Subsequent work will examine how multidirectional impacts, tissue composition, and individual anatomical variation influence local force transmission and injury risk, thereby extending the present findings to more realistic sport-specific loading conditions.

Taken together, these findings highlight the biomechanical plausibility of breast tissue injury under normal rugby tackling conditions and validate the capability of the developed wearable sensing system to capture such impacts. The consistent activation of sensors during tackling events, particularly those on the contact-side breast, provides strong evidence for the need to further investigate breast-specific injury risk and the potential role of protective equipment tailored for female athletes in collision sports.

### 3.8. Future Work and Broader Applications

#### 3.8.1. Overview, Applications and Potential Impacts

While the present study focused on the development and preliminary validation of a breast-mounted force sensing system for female athletes, the modular and flexible nature of the platform lends itself to broader applications across biomechanics, sports science, clinical diagnostics, and ergonomics.

In sports performance and injury prevention, similar sensor configurations have been employed for plantar pressure mapping and gait analysis, aiding in the early detection of pathological gait patterns, overuse injuries, and load asymmetries [90]. The localisation and scalability of the sensor array could enable use in joint-specific impact monitoring, such as the shoulder or lower back in high-risk sports, or skill-specific feedback systems—for example, measuring grip force in racket sports [91,92] or strike location in combat disciplines [93].

Beyond sports, there is potential for clinical applications in rehabilitation and care monitoring. The sensor array could be adapted for postural pressure assessment in wheelchair users or those spending long periods in bed, where real-time feedback is essential in pressure ulcer prevention [94]. Similarly, distributed skin-mounted FSRs have been shown to be effective in assessing interface pressures under orthotics, exoskeletons, or prosthetic devices, offering objective insights into fit and function [95,96], tight-fitting garments such as bras [97], pressure garments, and body armour.

The system also holds promise for use in ergonomic and occupational health contexts, such as evaluating load distribution during manual tasks or monitoring contact forces in wearable industrial exoskeletons [98]. With increasing interest in wearable human–machine interfaces (HMIs), the sensor array could be embedded into robotic or VR systems to detect user input and optimise actuator response [99,100].

#### 3.8.2. Leveraging AI for Enhanced Analysis

Building on the strong foundation of the portable breast impact monitoring platform, a critical next step is to harness artificial intelligence (AI) to enable intelligent pattern recognition and predictive analytics. Wearable sensor data in sports biomechanics has increasingly been augmented by machine learning (ML) and deep learning techniques—for example, in running gait analysis using inertial sensors and deep learning models such as CNNs and RNNs [101]. Similarly, broader reviews highlight how AI can enhance sports biomechanics research, from injury prediction to performance optimisation [102,103].

In the clinical and rehabilitation contexts, wearable sensors coupled with ML are already aiding in adaptive training and diagnostics involving human movement and recovery monitoring [104,105]. This trend is reinforced by reviews emphasising AI’s growing critical role in transforming healthcare with wearable sensing platforms [106]. Applied to the developed BIMS, integrating AI could enable real-time detection and classification of impact patterns, such as distinguishing between rugby tackles/activities and flagging potentially risky force profiles. Such automated insights could support coaches, sports scientists, and medical practitioners by informing safer training practices or protective equipment adjustments. This may in fact require integration of other sensors for reinforcement, such as motion sensors [8,107] or indeed bio/chemical sensing of sweat [11,12,108] for a more granulated source of information.

#### 3.8.3. Future Research Directions and Scalability

While the present work demonstrated both mechanical and preliminary field feasibility, we recognise the need for systematic validation under authentic sporting conditions. Future work will therefore focus on refining the wearable configuration, expanding testing to a larger and more diverse athlete cohort, and formalising the system’s application within protective equipment evaluation and injury-prevention frameworks. This will involve deploying the system during live training and match play to assess performance under full-intensity, multi-directional movement and breast deformation.

At present, direct on-field validity testing against a gold-standard instrument is not feasible, as no established reference system exists for quantifying localised breast impact forces under dynamic sporting conditions. The calibration procedures used in this study were therefore designed to replicate in situ testing conditions using a breast prosthesis in a controlled mechanical rig, allowing sensor performance to be characterised under realistic loading profiles. Future work could explore the development of more robust validation approaches, potentially integrating synchronised high-speed imaging or alternative mechanical surrogates to further enhance ecological validity and confidence in field data.

The integration of synchronised high-speed video footage and complementary physiological measurements will further refine force estimation and enhance robustness across real-world applications. Building on the system’s established calibration and verified sensitivity, the BIMS platform provides a foundation for standardised testing of breast protection devices and for field-based monitoring of athlete exposures during contact play. Further development of the capability of the custom drop rig (Section 2.3) to generate controlled and repeatable impact profiles, we propose a standardised methodology for laboratory testing of breast protective equipment. Using an instrumented breast phantom or prosthesis, protective gear can be assessed by comparing peak transmitted forces across impacts ranging from 500–1500 N, corresponding to submaximal to high-intensity real-world collisions. The percentage reduction in localised peak force measured by the BIMS array should serve as an objective attenuation index for evaluating and comparing protective materials and designs. This approach provides a reproducible framework for quantitative, evidence-based protective equipment validation.

To extend the system’s application to real-world sport, we recommend the deployment of the wireless BIMS platform in longitudinal field studies to quantify the magnitude, frequency, and spatial distribution of impacts experienced by athletes during training and competition. These data should be correlated with athlete-reported symptoms to determine critical force thresholds associated with pain or tissue injury, forming the basis for injury risk models. The same approach can also be used to compare the performance of different protective garments during actual play, establishing evidence-based criteria for protective efficacy.

Future work should explore these avenues through targeted trials, extending the current platform to larger athlete cohorts and real sporting environments. Because the system is built from readily available, off-the-shelf components, it can be scaled efficiently, enabling widespread data collection that can address currently unanswered research questions. This includes more robust characterisation of breast impact exposure and systematic assessment of the attenuation effects of commercially available or novel breast protective equipment. Increasingly, high-resolution video footage is available in both training and competition, and integrating sensor data with such footage would provide valuable context for interpreting impacts, refining estimations, and developing sport-specific injury risk models. In parallel, domain-specific calibration procedures and the development of context-aware algorithms will be essential to translate sensor data into actionable insights across different anatomical regions and population groups.

## 4. Conclusions

This work delivers the first validated, breast-mounted sensing system capable of quantifying localised impacts in high-intensity sport. By combining flexible thin-film sensors, a portable wireless platform, and rigorous calibration with controlled mechanical testing, the system provides reliable measures of both the magnitude and distribution of forces experienced by breast tissue. Crucially, it achieves this in a form that athletes can wear in realistic sporting conditions.

The ability to capture these measurements addresses a long-standing blind spot in sports science and athlete welfare. It creates, for the first time, a practical method to study breast impacts systematically, to evaluate protective equipment, and to inform design and policy decisions aimed at reducing injury risk. The platform also opens new avenues for research in health, medicine, and ergonomics wherever soft-tissue loading is a concern. In bridging engineering precision with sporting reality, this study establishes a foundation for future innovation in both scientific understanding and athlete protection.

## Figures and Tables

**Table 1:** Descriptions of breast coverage with respect to target sensor placement.

Left Breast Sensor ID	Right Breast Sensor ID	Coverage Description
8	16	The areola and nipple region are located where the mid-line and transverse line intersect
7	15	Between inferior quadrants and are located halfway between the mid-line and transverse line intersect and the inferior border
6	14	The inferior-medial quadrant and are located parallel to sensors 7 but 75% away from the mid-line towards the medial border
5	13	The medial quadrants and are located parallel to sensors 8/16 but 75% away from the mid-line towards the medial border
4	12	The superior medial quadrant and are located 50% away from the mid-line towards the medial border and 50% away from the transverse line towards the superior border
3	11	The superior lateral quadrant and are located 50% away from the mid-line towards the lateral border and 50% away from the transverse line towards the superior border
2	10	The lateral quadrants are located parallel to sensor 8 but 75% away from the mid-line towards the lateral border
1	9	The inferior-lateral quadrant and are located parallel to sensors 7/15 but 75% away from the mid-line towards the lateral border

**Table 2:** Participant characteristics.

Participant	Age (Years)	Height (cm)	Mass (kg)	Bra Size (AU Band & Cup)	Breast Size *
1	23	152	50	8A	1
2	24	170	110	18D	3
3	25	165	80	14C	2
4	50	158	68	14B	1

* Breast size ranked from 1 (small breasts, <350 mL per breast) to 4 (hypertrophic breasts, >1200 mL per breast) [81].

## Data Availability

The original contributions presented in this study are included in the article/Supplementary Materials. Further inquiries can be directed to the corresponding author.

## Supplementary Materials

The following supporting information can be downloaded at: https://www.mdpi.com/article/10.3390/s25216585/s1, Video S1: Over-the-ball breast impact heat maps, Video S2: Under-the-ball breast impact heat maps.

## Abbreviations

The following abbreviations are used in this manuscript:

FSR	Force Sensing Resistor
ADC	Analog to Digital Converter/Conversion
BLE	Bluetooth Low Energy
CAD	Computer Aided Design
$f_{s}$	Sampling Frequency
Sps	Samples per second
DAQ	Data Acquisition System
NRS	Numerical Ranking Scale
SLQ	Superior Lateral Quadrant
SMQ	Superior Medial Quadrant
ILQ	Inferior Lateral Quadrant
IMQ	Inferior Medial Quadrant
